# Absolute quantification of cassava brown streak virus mRNA by real-time qPCR

**DOI:** 10.1016/j.jviromet.2017.03.003

**Published:** 2017-07

**Authors:** Rudolph R. Shirima, Daniel G. Maeda, Edward Kanju, Gloria Ceasar, Flora I. Tibazarwa, James P. Legg

**Affiliations:** a International Institute of Tropical Agriculture, P. O. Box 34441, Dar es Salaam, Tanzania; b University of Dar es Salaam, P.O. Box 35179, Dar es Salaam, Tanzania; c Tanzania Commission for Science and Technology, P.O. Box 4302, Dar es Salaam, Tanzania

**Keywords:** Absolute quantification, CBSD, CBSV, UCBSV, Virus titre

## Abstract

Cassava brown streak disease (CBSD) is the most important virus disease of cassava and a major food security threat in Africa. Yearly economic losses of up to $100 million USD have been attributed to CBSD. The lack of information on plant-virus interactions has restricted progress in breeding for CBSD resistance. Virus quantification is becoming a major tool for the quick and reliable assessment of plant host resistance. Therefore, a protocol for specific absolute quantification of *Cassava brown streak virus* (CBSV) and *Ugandan cassava brown streak virus* (UCBSV) was developed. CBSV and UCBSV coat protein (CP) specific standard templates: CBSV (pFer2, 826 bp) and UCBSV (pUF1-R1-1, 732) respectively were generated and maintained in a TA cloning vector. These were used to construct standard curves using a TaqMan qPCR assay. Standard curves with acceptable amplification efficiencies (90–105%) and coefficients of determination (R^2^) greater than 0.99 were obtained. Infected cassava plants were sampled from a screenhouse and the field and used to validate this assay. Results obtained by testing several screenhouse and field samples revealed consistent absolute quantification assays for different CBSV and UCBSV isolates. This study presents the first protocol for absolute quantification of CBSVs and is expected to accelerate screening for CBSD resistance and hence breeding for CBSD resistance. The use of the method presented here should improve the clarity of virus quantification data as the results obtained are not influenced by varietal, host, seasonal or environmental conditions. Screening efficiency will also be greatly improved as there is no need for the use of reference genes consequently allowing for a larger number of samples to be analyzed. This will increase experimental precision in a timely and cost effective manner.

## Highlights

• First protocol for absolute quantification of cassava brown streak viruses.
• Standard templates for specific absolute quantification of CBSVs generated.
• Acceptable standard curves for specific absolute quantification of CBSVs prepared.
• Screening efficiency for CBSD-resistance sources will be greatly improved.

## 1 Introduction

Cassava brown streak disease (CBSD) is the most important virus disease of cassava and a major food security threat in Africa (Patil et al., 2015). Yield and storage root quality reductions of up to 70–100% have been reported (Hillocks et al., 2001), resulting in annual economic losses of up to US$ 100 million (Manyong et al., 2012, Ndunguru et al., 2015). In Tanzania alone, annual losses have been estimated at more than US$ 51 million (Ndyetabula et al., 2016). CBSD is known to be caused by two species of *Ipomoviruses* of the family Potyviridae: *Cassava brown streak virus* (CBSV) and *Ugandan cassava brown streak virus* (UCBSV) (Mbanzibwa et al., 2011; Winter et al., 2010). Recent reports show that CBSD is extending its range rapidly towards Central Africa and poses a major threat to cassava production in the region and neighbouring West African countries (Bigirimana et al., 2011, Legg et al., 2011, Mulimbi et al., 2012). Current approaches to contain CBSD include breeding for CBSD-resistant cassava varieties (Patil et al., 2015) as well as biotechnological methods such as the use of post-transcriptional gene silencing to generate transgenic varieties (Patil et al., 2011, Reddy et al., 2009). Other approaches include dissemination of clean planting material through community participation (Legg et al., 2015) and phytosanitary practices (Hillocks and Jennings, 2003, Storey, 1936).

Efforts to identify and exploit sources of resistance to CBSD started in the middle of the last century (Jennings, 1957, Jennings, 1960, Nichols, 1947). However, strong sources of resistance have yet to be identified in cassava varieties, although some are tolerant to the disease, as infected plants rarely develop necrotic rot symptoms in their tuberous roots (Hillocks and Jennings, 2003). An important facet of evaluating sources of resistance in on-going breeding work is determining the concentration of cassava brown streak viruses (CBSVs) in infected plants. Viral load quantification in cassava has recently gained importance, but of the few studies that have attempted this for CBSVs, all have been based on relative virus titre (Kaweesi et al., 2014, Mohammed et al., 2012, Moreno et al., 2011, Ogwok et al., 2015). Relative quantification results may be hard to interpret since they are estimated as fold changes in gene expression (Livak and Schmittgen, 2001) which requires an experimentally validated choice of reference genes (Schmittgen and Zakrajsek, 2000). Screening experiments are therefore best done using absolute quantification methods (Pfaffl, 2004). Furthermore, absolute quantitative PCR (qPCR) methods have been found to be more sensitive to gene expression variations caused by factors such as developmental and environmental variation (Lu et al., 2012), and the determination of absolute virus titre in cassava will greatly expand the potential for exploring the mechanisms of resistance and transmissibility of CBSVs. These techniques have been successfully used in other crops such as in studying the accumulation of viral load in the white-backed planthopper, Sogatella furcifera (Horvath), the vector of Southern rice black-streaked dwarf virus which affects rice production in China (An et al., 2015). In another example, Zhang et al., 2008, Zhang et al., 2010 report the detection of very low titres of Wheat dwarf virus and Rice stripe virus respectively in field collected samples. We present in this study an absolute quantification method for CBSVs in cassava which was achieved through developing a standard template from the coat protein region of published CBSV and UCBSV genome sequences and subsequently producing standard curves based on these standard templates using a modified version of the TaqMan assay protocol of Adams et al. (2013). This method should be of great importance to all those aiming to study the behavior of CBSVs in cassava plants and to use the new knowledge generated to select for improved resistance to these economically damaging viruses.

## 2 Material and methods

### 2.1 Primer sets for standard DNA template amplification and taqman RT-qPCR

Primers for standard template amplification were designed such that they anneal specifically to CBSV and UCBSV coat proteins (Table 1). Published CBSV and UCBSV genomes used for this purpose were obtained from NCBI [Accession numbers: HG965221, NC_012698, GU563327, FN434436, FN434437 and GQ329864 (CBSV); and NC_014791, HG965222, FN4343109, HM181930, FJ039520 and FJ185044 (UCBSV)] and aligned using CLC Main Workbench Version 7 (CLC bio, Qiagen). Primer and probe sequences used for real-time RT-qPCR were obtained from Adams et al. (2013) with modifications to the UCBSV specific forward primer (UCBSV CP-F-rev, Table 1). Primers for standard amplicons amplification were designed to flank those used in real-time RT-qPCR.

### 2.2 Preparation of standard DNA templates for standard curve construction

#### 2.2.1 Nucleic acid extraction

Total RNA was extracted from dried cassava leaves that had been preserved between sheets of plain newsprint held within wooden-framed plant herbaria. A cetyltrimetyl ammonium bromide (CTAB) total nucleic acid extraction protocol (Lodhi et al., 1994) optimized for cassava (Abarshi et al., 2010, Maruthi et al., 2002) with slight modifications was followed. Thirty-five mg of dried cassava leaf was placed in a 2 mL microcentrifuge tube for each sample separately, two DNase-/RNase-free sterile steel balls were added and the tube shaken on a GenoGrinder (SPEX SamplePrepP 2010) at 1500 rpm for 40 s. This procedure was repeated once to completely grind the leaf material into a fine powder. Tubes with leaf powder were transferred into a fume hood and 750 μL CTAB buffer was added (2.0%, w/v CTAB, 2.0 M NaCl, 2.0% PVP, 25 mM EDTA, 100 mM Tris-HCl pH 8.0, 0.2% fresh β-mercaptoethanol). Tubes were shaken vigorously on a vortex mixer and incubated at 65 °C for 15 min in a water bath. The nucleic acids were extracted by adding 750 μL chloroform:isoamyl mixture (24:1, Amresco), vortexed, centrifuged at 13.3 × *g* and 500 mL of the aqueous phase was transferred to a new sterile 1.5 mL microcentrifuge tube. The nucleic acids were precipitated in 0.6 vol (300 μL) of isopropanol (Amresco), incubated at −20 °C for 1 h and pelleted by centrifuging at 15.6 × *g*. The pellets were washed twice in 700 μL of 70% ethanol, centrifuged at 15.6 × *g* and the ethanol was discarded by decanting. The remaining ethanol was completely removed by pipetting and air drying for 30 min at room temperature. The pellets were dissolved in 100 μL of 1x tris-ethylene diamine tetraacetic acid (TE, Invitrogen) and nucleic acids allowed to re-suspend on ice for 30 min. The samples were treated with DNase1 to remove contaminating DNA according to the manufacturer’s instructions (Sigma). RNA quality and purity was determined by using a NanoDrop2000 spectrophotometer (Thermo Scientific) and used immediately for cDNA synthesis or stored at −80 °C for longer-term preservation.

#### 2.2.2 Two step RT-PCR

Complementary DNA (cDNA) was prepared from 1 μg of total RNA. The complete reverse transcriptase reaction (20 μL) contained 500 μM dNTPs (Thermo Scientific), 50 mM oligo dT_18_ (New England Biolabs), 1x M-MLV buffer (Sigma), 200 units M-MLV reverse transcriptase (RT) (Sigma) and milliQ water up to 20 μL.

A portion of this reaction containing only dNTPs, oligo dT_18_ (New England Biolabs) and total RNA and was first heated at 70 °C for 5 min and immediately chilled on ice prior to the addition of the remaining components, a mixture containing 1x M-MLV RT buffer and M-MLV reverse transcriptase (RT) (Sigma) and the complete reaction was incubated at 37 °C for 50 min followed by M-MLV RT deactivation at 80 °C for 20 min. No reverse transcriptase and a reaction containing PCR water instead of RNA were included as negative controls. The cDNA was placed on ice for immediate use or stored at −20 °C for future use. Primers (Table 1) specific CP portions of CBSV and UCBSV viruses were used to amplify amplicons to be used as standard templates in absolute quantification assays. The PCR reaction contained 1 μL cDNA, 1x PCR buffer (Sigma), 2 mM MgCl_2_ (Sigma), 200 μM dNTPs (Thermo Scientifc), 0.625 units Taq DNA polymerase (Sigma) and either 250 μM (each of CBSV-CP-Fer2 or CBSV-CP-Fer3 and CBSV-CP-R1-1 primer pairs) or 200 μM (each of UCBSV-CP-F1 or UCBSV-CP-F1-1 and UCBSV-CP-R1-1). The PCR program included 95 °C for 3 min initial denaturation, 35 PCR cycles of 94 °C for 20 s, 52 °C for 40 s and 72 °C for 50 s and a final extension step at 72 °C for 10 min.

#### 2.2.3 Cloning, transformation of bacteria and plasmid isolation

Gel purified or fresh PCR products were TA cloned into the pCRII-TOPO vector (Invitrogen) according to the manufacturer’s instructions and transformed into the TOP10 competent *Escherichia coli* (Invitrogen) and plated onto LB plates according to the manufacturer’s instructions. Transformants were screened by selecting white colonies and performing colony PCR using M13 primers and one of the CBSV or UCBSV CP specific primers (Table 1). Corresponding colonies were cultured overnight in liquid LB medium supplemented with 50 μg/mL ampicillin. Plasmid isolation was achieved using the PureLink Quick Plasmid DNA Miniprep Kit (Invitrogen) and DNA was quantified using a NanoDrop2000 spectrophotometer (Thermo Scientific). Confirmation of the cloned CBSV and UCBSV CP sequences was done by sequencing some of the purified plasmids. Purified plasmids were sent to the Macrogen, USA sequencing service provider. Raw sequences were assembled and edited using CLC Main Workbench Version 7. Phylogenetic analyses were run using the Maximum Likelihood algorithm to estimate evolutionary distances (Kimura, 1980) in MEGA6 bioinformatics software.

### 2.3 Real-time RT-qPCR verification of standard templates and construction of standard curves

Plasmids carrying CBSV or UCBSV CP inserts were linearized using the *BamH1* restriction site located downstream of the insertion site. It is recommended to linearize plasmids before qPCR to avoid overestimation of the obtained results due to presence of supercoiled conformations (Chen et al., 2007, Hou et al., 2010). Plasmid copy numbers were determined using the Avogadro’s constant (6.022141 × 10^23^) in Eq. (1). Optimal working concentrations were prepared for each plasmid; 3.9382E + 06 copies/ng (**pFer2**, CBSV) and 9.8455E + 06 copies/ng (**pUF1R1-1**, UCBSV) and serial dilutions were made to produce a tenfold six-point dilution series (Table 2). TaqMan real-time qPCR assays were performed using CBSV and UCBSV specific primers and probes (Adams et al., 2013; Table 1). The qPCR reaction contained 1 x PCR buffer, 4.5 mM MgCl_2_, 0.45 mM dNTPs, 100 nM probe, 30 nM ROX solution (Thermo Scientific) and 0.625 units of *Taq* DNA polymerase (all core reagents from Sigma). For the CBSV standard curve, 300 nM each of the forward and reverse primer was used, while 150 nM primer was used in the UCBSV qPCR reaction. Primers and probes were manufactured by Integrated DNA Technologies, Belgium. A total qPCR reaction mixture of 25 μL was achieved by the addition of PCR grade water and 2 or 5 μL of linearized plasmid (Table 2) for the CBSV and UCBSV standard curves respectively. The qPCR reaction was run on a Stratagene real-time PCR instrument (Mx3000P, Agilent Technologies). Each reaction was run in duplicate with the thermo cycling profile comprising 10 min initial denaturation at 95 °C and 40 cycles of 95 °C for 15 s and 60 °C for 60 s with fluorescent data collection during the 60 °C step. Data acquisition was done by MxPro Real-time QPCR software, version 4. A set of cDNA samples was run with cytochrome oxidase1 (COX) specific primers and probes (Table 1) as an internal positive control, a sample with known CBSV or UCBSV infection and no-template control with PCR water in place of linearized plasmid.

(1) $$Plasmidcopynumber=\frac{6.023\times 10^{23}\times plasmidamount\left(ng\right)*}{MWx1x10^{9}}$$

Where MW = plasmid molecular weight, (=plasmid size (bp) x molar mass per base (650 g mol^−1^ bp^−1^)

6.023 × 10^23^ molecules/mole = Avogadro’s constant

*Plasmid amount was calculated from plasmid concentration determined by NanoDrop2000 spectrophotometer

### 2.4 Validation of absolute quantification assay

#### 2.4.1 Source of CBSVs-infected plants

Cassava leaflets were sampled from field-collected cassava samples preserved in herbaria. Some of these had been collected from Bagamoyo (Tanzania), Bujumbura (Burundi) and South Kivu (eastern Democratic Republic of Congo). Samples from Bagamoyo were picked from batches collected at three time points and the CBSVs species detected were identified. All samples used for this purpose were collected between January and December, 2015. Samples with CBSV were collected at two, three and six months after planting (MAP) while samples infected with UCBSV were collected at four, five and seven MAP. Samples from Bujumbura and South Kivu came from farmers’ fields with varying crop age between three and ten MAP. In addition, leaf samples were also picked from screenhouse-maintained (IITA, Dar es Salaam) virus cultures with CBSV or UCBSV single infections.

#### 2.4.2 Absolute quantification of CBSVs titre using TaqMan qRT-PCR

Identification of virus species was achieved by using a real-time RT-PCR TaqMan assay (Adams et al., 2013). Samples with the appropriate virus identity were analyzed by an absolute quantification qPCR reaction set up using a TaqMan assay for specific quantitation of CBSV and UCBSV. Each standard reaction was duplicated and run concurrently with duplicate test cDNA samples. A set of cDNA samples was run with cytochrome oxidase 1 specific probes as an internal positive control assay running concurrently on the same reaction plate. Reactions were performed with the Stratagene Mx3000P instrument and the Mx3000P qPCR software was used for the data acquisition described.

## 3 Data analysis

Absolute quantification of CBSV and UCBSV was determined by running the default settings of the MxPro qPCR software on the Stratagene Mx3000P qPCR system (Agilent Technologies). The data obtained were assembled in Microsoft Excel and correlation analysis performed using the Statistical Analysis System (SAS Institute Inc., Cary, NC, USA, version 9.3). Molecular Phylogenetic analyses for CBSV and UCBSV CP sequences were done using the Maximum Likelihood method in MEGA6 (Tamura et al., 2013).

## 4 Results

Four pairs of primers, two each for CBSV and UCBSV were designed to specifically amplify fragments of the CBSVs coat protein. Resulting PCR products of the expected size were TA cloned into appropriate vectors and transformed into suitable *E. coli* competent cells. Plasmids were finally recovered, linearized and used to construct standard curves. These standard curves were used to optimize absolute quantification assays for CBSVs in nucleic acids extracted from CBSVs-infected cassava.

### 4.1 Primer specificity

**Fig. 1:**
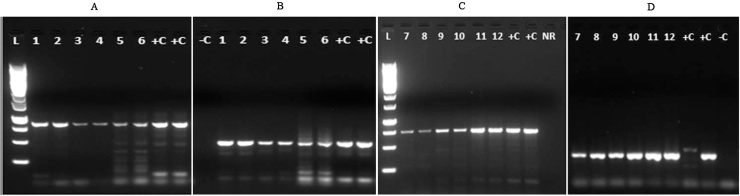
Amplification of CBSV and UCBSV coat protein sequences. Letters represent the primer pair used (A, CBSV-CP-Fer2/CBSV-CP-R1-1; B, CBSV-CP-Fer3/CBSV-CP-R1-1; C, UCBSV-CP-F1-1/UCBSV-CP-R1-1; D, UCBSV-CP-F1/UCBSV-CP-R1-1. L = GeneRuler 1 kb DNA Ladder, Thermo Scientific. −C = negative control sample (PCR water), +C = positive control, NR = no RT (reverse transcriptase excluded), 1–6 = cassava leaf samples obtained from CBSV (A, B) infected plants, 7–12 = cassava leaf samples obtained from UCBSV (C, D) infected plants.

CBSV and UCBSV coat protein specific primers were used to amplify PCR products of 826 bp (CBSV-CP-Fer2/CBSV-CP-R1-1), 447 bp (CBSV-CP-Fer3/CBSV-CP-R1-1), 440 bp (UCBSV-CP-F1-1/UCBSV-CP-R1-1) and 732 bp (UCBSV-CP-F1/UCBSV-CP-R1-1). Amplicons of the expected size were obtained (Fig. 1).

### 4.2 Cloning, transformation and DNA sequencing

**Fig. 2:**
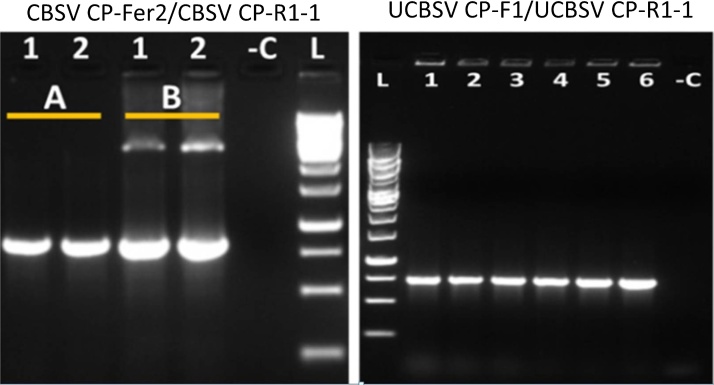
Colony PCR of transformed *E. coli*. CBSV and UCBSV specific primers were used to amplify the appropriate cloned coat protein fragments. Primers used are indicated on the top of the gel picture. A – represents bacteria colony, B – represents corresponding purified plasmids of the bacteria colony 1 and 2, the numbers 1–3 = bacterial colony, 4–5 = purified plasmid, 6 = positive control, L = 1 Kb DNA ladder, −C = negative control (PCR water).

**Fig. 3:**
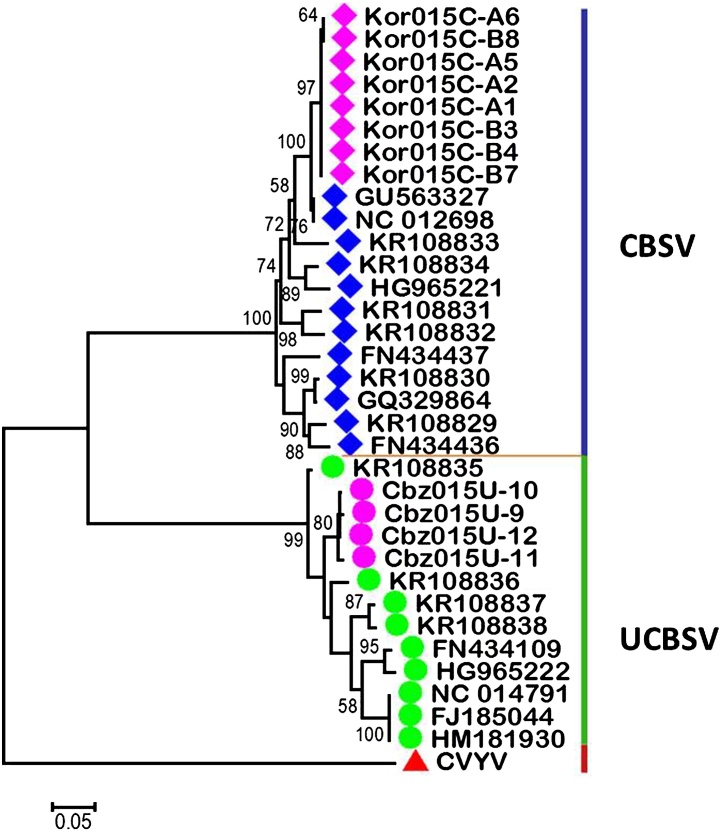
Molecular Phylogenetic analysis for CBSV and UCBSV CP sequences by the Maximum Likelihood method (1000 bootstraps). The evolutionary history was inferred employing the Kimura 2-parameter model (Kimura, 1980) where 34 nucleotide sequences were analyzed including 12 new CBSVs CP sequences. Evolutionary analyses were conducted in MEGA6 (Tamura et al., 2013). CBSV CP sequences, this study. Published CBSV sequences. UCBSV CP sequences, this study. Published UCBSV sequences. Cucumber vein yellowing virus.

Transformed bacteria were screened by colony PCR and presence of the insert was confirmed using M13 forward and or M13 reverse primers with the appropriate CBSV or UCBSV specific primer. Corresponding purified plasmids for the CBSV plasmid (pFer2) and cDNA synthesized from the original RNA sample were also included during the colony PCR run. Only results from colony PCR are shown for the UCBSV transformants (pUF1-R1-1). Results revealed the presence of the appropriate CBSV CP insert (Fig. 2). Results from restriction analysis (data not shown) of the colonies also confirmed the identity of the inserted CP fragments. PCR water was used as a negative control. Further confirmation of the cloned CBSVs CP fragments was done by sequencing the plasmids. Phylogenetic analyses of the resulting sequences showed that these sequences clustered in the appropriate CBSV and UCBSV species groups (Fig. 3).

### 4.3 Standard curves and qPCR amplification efficiency

**Fig. 4:**
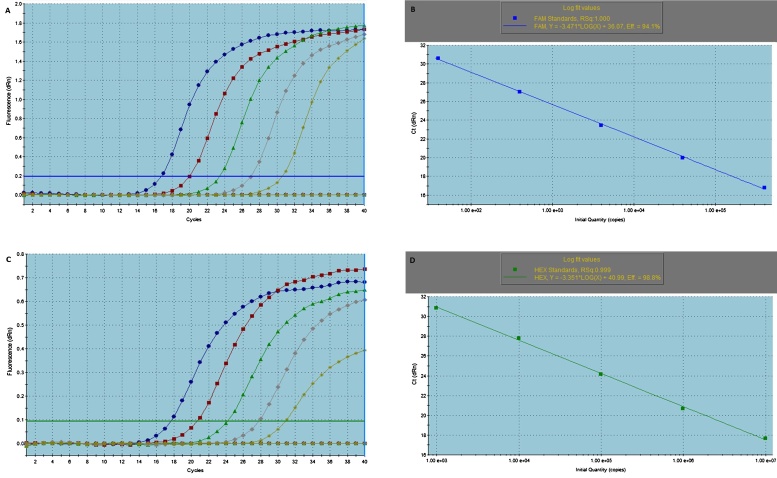
PCR amplification plots and standard curves of serially diluted linearized plasmids obtained using TaqMan assay probe chemistry. A, amplification plots of CBSV clone (pFer2); B, standard curve of CBSV clone (pFer2); C, amplification plots of UCBSV clone (pUF1R1-1); D, standard curve of UCBSV clone (pUF1R1-1). Amplification efficiencies of 94.1 and 98.8 and coefficients of correlation equal to 1 and 0.999 were achieved for CBSV and UCBSV absolute quantification respectively.

Absolute quantification assays were set up using TaqMan probe chemistry. Serial dilutions of purified and linearized plasmids (pFer2 and pUF1R1-1, Table 2) were used to run CBSV or UCBSV specific qPCR assays in duplicate reactions. Standard curves were generated with PCR amplification efficiencies between 94 and 99% and coefficients of correlation (Rsq) greater than 0.998 (Fig. 4).

### 4.4 Validation of absolute quantification assay for determination of virus titre in CBSVs infected cassava

**Fig. 5:**
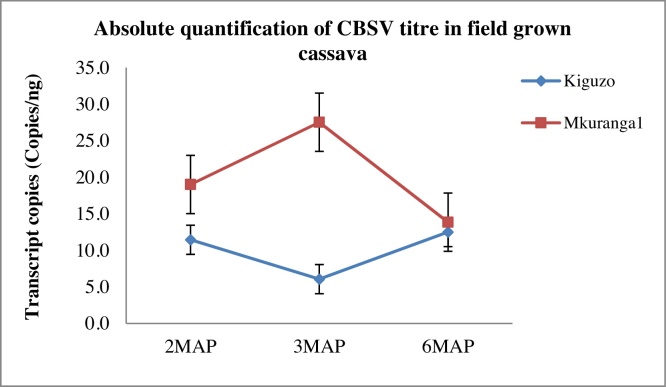
Absolute quantification of CBSV over three sample collection points in time. 2MAP = June, 3MAP = July, 6MAP = October.

**Fig. 6:**
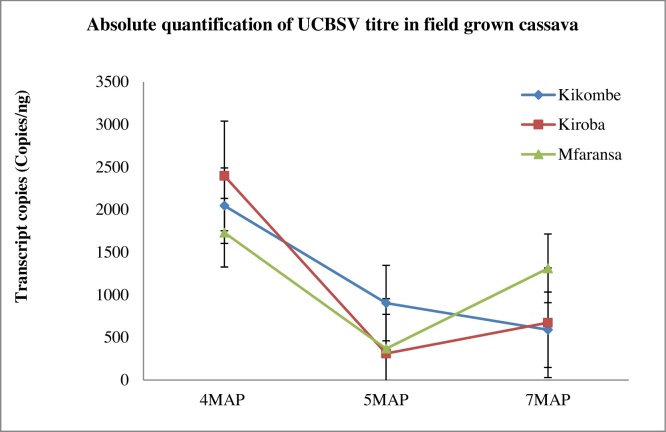
Absolute quantification of UCBSV over three sample collection points. 4MAP = July, 5MAP = August, 7MAP = November.

Three samples per variety were collected from CBSVs-infected cassava plants at two, three and six MAP. A central leaflet was picked from the third leaf counting from the top most fully expanded leaf. Absolute quantification assays were run for each of these and the mean CBSV titre (copies/ng) was determined. Two and three varieties per analysis were selected for the absolute quantification of CBSV and UCBSV respectively at three different sampling points in time. The varieties: Kiguzo and Mkuranga1 were analyzed for CBSV titre while Kikombe, Kiroba and Mfaransa were analyzed for UCBSV titre. The selection of samples was based on the availability of CBSV or UCBSV infected plants at each of the selected sampling points. Relatively low mean absolute CBSV titres were observed for both varieties analyzed for CBSV titre; 10 and 20.1 copies/ng for Kiguzo and Mkuranga1 respectively. No clear CBSV concentration change patterns were observed with respect to the two, three and six MAP sampling points tested (Fig. 5). However, CBSV titre increased about 1.4-fold and decreased about 1.9-fold at 3MAP for Mkuranga1 and Kiguzo respectively. Quantification of UCBSV however revealed higher virus titres as compared to the CBSV titres. Up to 2396 copies/ng were observed for Kiroba followed by Kikombe (2047 copies/ng) and Mfaransa (1730 copies/ng) at 4MAP. There was a marked overall decrease in UCBSV concentration at 5MAP and a slight increase at 7MAP for Mfaransa and Kiroba although UCBSV titre decreased further for Kikombe (Fig. 6) when the varieties were treated individually.

Samples collected from screenhouse maintained plants had comparatively high virus titre with a mean CBSV titre of 2.05E + 03 copies/ng for the variety Kiguzo and 3.29E + 05 for Rasta. Samples collected from South Kivu, DRC and Bujumbura had only UCBSV and analysis of absolute virus titre showed a higher mean UCBSV titre in South Kivu (1.55E + 04 copies/ng) as compared to Bujumbura (2.19E + 02 copies/ng) (Table 3). Amplification efficiencies of between 90 and 103% and coefficients of determination (R^2^) > 0.998 were obtained supporting reliable quantification results. Other aspects of the quantification assays such as cycle threshold, Ct values, standard curve slopes and number of samples analyzed are also presented in Table 3. UCBSV was quantified in UCBSV-infected samples from South Kivu DRC and the CBSD severity and UCBSV titre compared (Table 4). Sixty-six samples were analyzed that were obtained from five cassava varieties. Mean UCBSV titre was calculated from at least eight plants for each of these varieties. There was no significant correlation between UCBSV titre and symptom severity for these samples, however, symptom severities were in all cases similar, ranging from 2.7 to 3.0. Additionally, a weak negative correlation (r = −0.04031, p < 0.05) was also observed for CBSD incidence in all of these five varieties.

## 5 Discussion

The most important economic losses in East Africa’s cassava production are attributed to CBSD. Effective management of the disease requires concerted efforts in breeding for resistance. Enhancing the resistance of cassava to CBSD is currently being done using the techniques of conventional breeding, marker-assisted selection and genetic engineering, whilst responses to viruses are assessed using field-based phenotyping, testing for virus presence/absence and determination of relative virus concentration. Overall, these methods fail to cope with the speed required to screen the growing number of cassava clones and or varieties with potential CBSD resistance sources. One of the main problems with the use of methods to assess relative virus concentration is the inherent limitation of the need for specific reference genes (Schmittgen and Zakrajsek, 2000). In this study, a protocol for absolute quantification of CBSVs by using virus-specific standard curves was developed in order to make virus assessments quicker and more informative.

In this paper, we report the successful development of a protocol for determining the absolute virus concentration of the two cassava brown streak virus species: CBSV and UCBSV. Standard curves were generated from CBSV and UCBSV CP-specific standard templates that had been cloned and maintained in a TA cloning vector which allowed direct cloning of PCR products. This choice of vector saved time by excluding linearizing of the vector and the cost that would have been incurred to purchase restriction enzymes. Phylogenetic analyses revealed clustering of the cloned CBSV and UCBSV standard templates with the sequences of CBSV and UCBSV published in GenBank. The CBSV standard template (pFer2) with sequences named Kor015C-A1, A2, A3, A5, A6, B3, B4, B7 and B8 shared greatest identity (99%) with a CBSV isolate from Korogwe, Tanzania (GenBank accession # GU563327). The UCBSV standard template (pUF1R1-1) with sequences named Cbz015U-9, 10, 11 and 12, shared greatest identity (97%) with a published UCBSV isolate, also from Tanzania (GenBank accession #KF878103). These results confirm the identity and integrity of the standard templates used in this study. The use of appropriate standard templates in generating standard curves is an important requirement for obtaining accurate absolute quantification data. Cloned DNA is very stable and therefore gives reliable results as it generates highly reproducible standard curves (Pfaffl, 2004). It is even more appropriate when standard curves are generated from nucleic acid targets obtained from the same species as the test material as these are amplified with equivalent efficiencies ensuring reliable quantification data. In this study, standard templates specific to the target viruses to be quantified (CBSV and USBSV respectively) were generated.

TaqMan qPCR assays were employed to perform the absolute quantification CBSVs in this study. TaqMan assays are highly sensitive and specific, therefore it is important to use high precision pipettes. As opposed to absolute quantification, the use of relative quantification methods must be carefully assessed to avoid false negative or false positive results that may result from the influence of other factors such as environmental variation or inappropriate reference genes (Lu et al., 2012). It should be noted, however, that optimal thermal cycling conditions must be critically evaluated. The accuracy of the standard dilutions is paramount in obtaining reliable standard curves. Standard curves used in this study were optimized to obtain acceptable amplification efficiencies of between 90 and 105% and coefficients of determination greater than 0.99 (Taylor et al., 2015).

Few crop research studies have reported using absolute quantification of viral load. One such study, however, is the quantification of rice stripe virus in rice and in the small brown planthopper (Zhang et al., 2008) using virus-specific standard templates in TaqMan assays. In a second example, Zhang et al. (2010) detected very low concentrations of wheat dwarf virus. More recently, the accumulation of viral load in the white-backed planthopper, *Sogatella furcifera* (Horvath), the vector of Southern rice black-streaked dwarf virus which affects rice production in China was reported (An et al., 2015). One of the major characteristics of a resistant plant is being able to restrict virus loads to low levels (Maruthi et al., 2014). The absolute quantification protocol for CBSVs presented here will therefore be very useful in identifying such cassava varieties within the several studies that are currently being conducted in East and Central Africa, thereby speeding up the search for CBSD-resistant cassava. Realizing that there are several factors that need to be resolved before a true CBSD-resistant cassava is obtained, we emphasize the importance of employing intensive field research coupled with high quality screening methods such as the one presented in this study in order to fully exploit the advantages of host plant resistance in combating CBSD. The use of such tools in promoting the development of durable resistance has been pinpointed as crucial for the sustainable management of pathogens and pests (Mundt, 2014). We present this method in anticipation that it will be adopted to speed up the screening of large numbers of cassava genotypes in the search for sources of CBSD resistance. Recent contributions towards this goal have included the development and application of relative virus titre methods (Kaweesi et al., 2014, Maruthi et al., 2014, Ogwok et al., 2015). Results from these studies showed that: (i) virus titre varied between cassava varieties, within plants and over time; (ii) a particular variety could not be consistently classified with a particular virus resistance level between different studies or growth conditions. In all cases, relatively small numbers of samples were examined, and where comparisons were made between field-grown varieties, these were restricted to a single location. These observations highlight the need for screening large numbers of samples and genotypes collected from diverse environments in order to draw the appropriate conclusions about interactions between the CBSVs and their cassava host. Whilst relative quantification-based assessments might be initially easier and cheaper to set up, the absolute quantification method presented here has the following important advantages. Firstly, absolute quantification eliminates the use of reference genes which can be difficult to select (Schmittgen and Zakrajsek, 2000) and whose expression may vary among tissues/organs and may change due to environmental conditions (Silver et al., 2006) therefore giving misleading results. Reference point selection in time-course studies may also be difficult to achieve, especially when working in the field where situations are very dynamic. Secondly, absolute quantification focuses entirely on the virus, thus simplifying the interpretation of results as this avoids the influence of other factors. Thirdly, this protocol is much easier to optimize and scale out to other crop/virus pathosystems. Finally, standard templates are preserved in plasmids where they are stable and can be stored for long periods. Colonies with the appropriate inserts can be cryopreserved and plated when required and this reduces the cost of standard template preparation.

To validate the method presented here, a number of samples from different locations and cassava varieties were tested. Analysis of a set of CBSV and UCBSV infected cassava varieties grown in the screenhouse or in the field showed that quantified CBSV concentration was lower than UCBSV concentration in single infections. The relatively comparable UCBSV titres across all samples tested in this study suggest that there is a fairly stable distribution of UCBSV across different infected plant populations. We observed an overall stable CBSV titre in two varieties (Kiguzo and Mkuranga1) from 2 MAP to 6MAP, although Mkuranga1 had a slight increase while Kiguzo had a slight decrease at 6MAP. In contrast, the titre of UCBSV dropped sharply from 4MAP to 5MAP and increased slightly at 7MAP for all tested varieties suggesting that plant growth stage was a more important determinant of virus titre than cassava genotype for these particular varieties. This observation suggests that properly analyzed virus titre data at different plant growth points will provide a better understanding of the potential of a given genotype/variety for use as a source of CBSD resistance. However, further investigation with different infection and virus types is required to confirm these observations and improve understanding of virus-host interactions over time. The absence of an obvious correlation between virus titre and severity of CBSD foliar symptom expression was comparable to previous reports (Kaweesi et al., 2014, Ogwok et al., 2015), although future studies will need to examine plants with more strongly divergent symptom phenotypes – ranging from mild to very severe. Such work should also aim to determine the relationship between virus titre and impacts on plant growth and yield. Froissart et al. (2010) reviewed several studies that examined the link between virus titre and virulence but were unable to make clear conclusions, partly since most of the reviewed work correlated virulence to disease symptoms, but also since small sample numbers were considered in most of the studies. In order to draw robust conclusions about the factors that determine host resistance and therefore come up with sustainable solutions for cassava farmers, we therefore suggest that more intensive research should be carried out to study the mechanisms of infection in relation to virus titre, virulence versus host response to infection. A key requirement for such research is that it makes use of a diverse set of samples obtained from contrasting cassava genotypes planted at multiple locations with varying virus inoculum pressure.

## 6 Conclusion

Cassava has been reported as the most climate resilient crop (Jarvis et al., 2012) and is therefore critical for the future food security of Africa, which is the continent where production of this crop is greatest. Screening for disease resistance is an important facet in the management of plant viruses. CBSD is the most important cassava virus disease in East Africa, and there are no strongly resistant varieties identified so far. Most of the cassava landraces are susceptible to CBSD, and many have been wiped out in regions to which CBSD has recently spread – notably the Great Lakes region of East and Central Africa. This means that there is already a significant loss of genetic diversity due to CBSD thereby depriving the scientific community of resources for future crop improvement. Screening for resistance is a key component of CBSD management programmes. Existing screening methods are expensive due to the limitation in the number of samples that can be analyzed. We have therefore developed a protocol for absolute quantification of CBSVs which if adopted will greatly speed up the screening of germplasm already in breeding programmes in the region. Rigorous screening experiments however are required in order to clearly understand the mechanisms of resistance. It is clear from the results obtained in this study that plant resistance mechanisms cannot be fully exploited unless promising germplasm is screened through the entire crop cycle. Out-scaling of the methods presented here to other root and tuber crops that are equally affected by viruses will also greatly strengthen the management programmes already in place.

## Figures and Tables

**Table 1:** Primer pairs and probes targeting CBSVs coat protein and used for RT-PCR and real-time RT-qPCR in this study.

Primer name^c^	Sequence (5′ to 3′)^d^	Annealing site^e^
CBSV-CP-Fer2^a^	GAAGGGATTGGAYTRGAAGGA	7390–7410
CBSV-CP-R1-1^a^	GAACGCGGTATCCACACATA	8197–8216
CBSV-CP-Fer3^a^	AAGCAATTGAYAARGATGAGA	7739–7759
UCBSV-CP-F1−1^a^	AGAGATCTGGAAAGGAAGT	7981–7999
UCBSV-CP-R1-1^a^	CTCGCCAYGACTTCTCATT	8403–8421
UCBSV-CP-F1^a^	GTGARGCAAGAGRAGAAGA	7689–7707
CBSV CP-F^b^	GCCAACTARAACTCGAAGTCCATT	
CBSV CP-probe^b^	[FAM]-AGTCAAGGAGGCTTCGTGCYCCTC-[BHQ1]	
CBSV CP-R^b^	TTCAGTTGTTTAAGCAGTTCGTTCA	
UCBSV CP-F-rev^a^	AGATYAAGAARACDTTCAAGCCTCCAA	8119–8145
UCBSV CP-probe^b^	[HEX]-TCAGCTTACATTTGGATTCCACGCTCTCA-[BHQ1]	
UCBSV CP-R^b^	AATTACATCAGGRGTTAGRTTRTCCCTT	
COX-F^b^	CGTCGCATTCCAGATTATCCA	
COX probe^b^	[HEX]-AGGGCATTCCATCCAGCGTAAGCA-[BHQ1]	
COX-R^b^	CAACTACGGATATATAAGRRCCRRAACTG	

a Primers developed or modified in this study.

b Primers and probes used according to Adams et al. (2013).

c Designations with −F, −Fer denote forward primers and those with −R denote reverse primers, those with −probe represent TaqMan probes.

d R represents A or G; Y represents C or T; D represents A, G or T.

e Residue numbers refer to sequences of *Cassava brown streak virus* (GQ329864) and *Ugandan cassava brown streak virus* (FN434109).

**Table 2:** Serial dilutions of linearized plasmid.

CBSV standard template (pFer2)				
Dilution^#^	Copy number (Copies/ng)^*^	Mass of plasmid required	Final concentration of plasmid (g/μL)	Volume used for qPCR (μL)
0	**3.9382E** **+** **06**	2.0714E-11	1.0357E-11	**2**
1	3.9382E + 05	2.0714E-12	1.0357E-12	
2	3.9382E + 04	2.0714E-13	1.0357E-13	
3	3.9382E + 03	2.0714E-14	1.0357E-14	
4	3.9382E + 02	2.0714E-15	1.0357E-15	
5	3.9382E + 01	2.0714E-16	1.0357E-16	

UCBSV standard template (pUF1R1-1)				
0	**9.8455E** **+** **06**	5.08E-11	1.02E-11	**5**
1	9.8455E + 05	5.08E-12	1.02E-12	
2	9.8455E + 04	5.08E-13	1.02E-13	
3	9.8455E + 03	5.08E-14	1.02E-14	
4	9.8455E + 02	5.08E-15	1.02E-15	
5	9.8455E + 01	5.08E-16	1.02E-16	

Linearized plasmids were quantified using a NanoDrop spectrophotometer. ^*^Plasmid copy numbers were determined and diluted serially to obtain six ^#^serial dilution points at 10^00^ (undiluted), 10^−1^, 10^−2^, 10^−3^, 10^−4^ and 10^−5^.

**Table 3:** Absolute quantification of CBSV and UCBSV in screenhouse and field-collected cassava samples.

Site	N	Virus assay	Threshold (dRn)	Ct (dRn)	aCBSVs titre (copies/ng)	bRSq (dRn)	Slope (dRn)	bEff. (%)
IITA Screenhouse	28	CBSV	0.3359	27.20	2.05E + 03	0.998	−3.484	95.0
IITA Screenhouse	28	UCBSV	0.177	24.60	3.29E + 05	1	−3.489	93.5
Bagamoyo, Tanzania	36	CBSV	0.0933	32.50	2.35E + 01	0.997	−3.558	91.0
Bagamoyo, Tanzania	27	UCBSV	0.0653	32.32	1.15E + 03	0.998	−3.397	97.0
South Kivu, DRC	66	UCBSV	0.0567	30.10	1.55E + 04	0.997	−3.269	102.3
Bujumbura	12	UCBSV	0.0653	30.10	2.19E + 02	0.998	−3.397	97.0

a Calculation of plasmid copy numbers used to produce standard curves for absolute quantification of CBSVs was achieved using Eq. (1). Mean values presented were calculated from sets of given sample numbers (N).

b Amplification efficiencies between 90 and 103% and coefficients of correlation greater than 0.998 are considered to be indicative of a successful qPCR assay.

**Table 4:** Absolute quantification of UCBSV in cassava samples collected from South Kivu, DRC.

N	Variety	Ct (dRn)	UCBSV titre (copies/ng)	aCBSD severity	CBSD incidence (%)
14	Local	29.5	2.71E + 04	2.9	72.1
13	Msikiilo	30.5	2.12E + 04	2.7	26.7
12	Mvuama	28.3	4.27E + 04	2.9	16.1
8	Naeunde	32.8	2.69E + 03	3.0	34.4
19	Sawasawa	29.6	2.34E + 04	2.9	77.4

a CBSD foliar symptoms were recorded on 30 plants per field using a scale of 1–5, where 1 = healthy asymptomatic and 5 = the most severe symptoms: mean severity scores were calculated using only symptomatic plants with scores 2–5; **N** = number of fields sampled per variety.
